# *De novo* Sequence Assembly and Characterization of *Lycoris aurea* Transcriptome Using GS FLX Titanium Platform of 454 Pyrosequencing

**DOI:** 10.1371/journal.pone.0060449

**Published:** 2013-04-09

**Authors:** Ren Wang, Sheng Xu, Yumei Jiang, Jingwei Jiang, Xiaodan Li, Lijian Liang, Jia He, Feng Peng, Bing Xia

**Affiliations:** 1 Institute of Botany, Jiangsu Province & Chinese Academy of Sciences, Nanjing, China; 2 Bioinformatic Center, Nanjing Agricultural University, Nanjing, China; Auburn University, United States of America

## Abstract

**Background:**

*Lycoris aurea*, also called Golden Magic Lily, is an ornamentally and medicinally important species of the Amaryllidaceae family. To date, the sequencing of its whole genome is unavailable as a non-model organism. Transcriptomic information is also scarce for this species. In this study, we performed *de novo* transcriptome sequencing to produce the first comprehensive expressed sequence tag (EST) dataset for *L. aurea* using high-throughput sequencing technology.

**Methodology and Principal Findings:**

Total RNA was isolated from leaves with sodium nitroprusside (SNP), salicylic acid (SA), or methyl jasmonate (MeJA) treatment, stems, and flowers at the bud, blooming, and wilting stages. Equal quantities of RNA from each tissue and stage were pooled to construct a cDNA library. Using 454 pyrosequencing technology, a total of 937,990 high quality reads (308.63 Mb) with an average read length of 329 bp were generated. Clustering and assembly of these reads produced a non-redundant set of 141,111 unique sequences, comprising 24,604 contigs and 116,507 singletons. All of the unique sequences were involved in the biological process, cellular component and molecular function categories by GO analysis. Potential genes and their functions were predicted by KEGG pathway mapping and COG analysis. Based on our sequence analysis and published literatures, many putative genes involved in Amaryllidaceae alkaloids synthesis, including *PAL*, *TYDC OMT*, *NMT*, *P450*, and other potentially important candidate genes, were identified for the first time in this *Lycoris*. Furthermore, 6,386 SSRs and 18,107 high-confidence SNPs were identified in this EST dataset.

**Conclusions:**

The transcriptome provides an invaluable new data for a functional genomics resource and future biological research in *L. aurea*. The molecular markers identified in this study will provide a material basis for future genetic linkage and quantitative trait loci analyses, and will provide useful information for functional genomic research in future.

## Introduction

The genus *Lycoris* is an important group of Amaryllidaceae composed of approximately 20 species of flowering plants native to the moist warm temperate woodlands of eastern and southern Asia, of which 15 (10 endemic) are distributed in China. Most of the *Lycoris* species are commonly cultivated in China, Korea, Japan and Vietnam as bulbous plants [1], [2]. In comparison with other well-known bulb flowers, such as narcissi and lilies, *Lycoris* has its own characteristics and merits. It comes into flower at a time when few other bulbous plants are active. The flowers are characterized by their pastel and plentiful colors as well as by beautiful and varied shapes [1]. So the *Lycoris* species are all very popular with considerable acceptance as ornamental plant [3] and most of them have been successfully cultivated. In the past several decades, some of the *Lycoris* species, cultivars, and hybrids such as *Lycoris radiata* and *Lycoris aurea* have been used worldwide. Meanwhile, the demand for *Lycoris* as a commercial horticultural product has been increasing steadily, so the breeding of varieties with new flower forms and/or colors has become desirable for *Lycoris*.

Moreover, *Lycoris* species are all of medical values. The bulbs of *Lycoris* have been used in traditional Chinese medicine (TCM) for a long time and some Amaryllidaceae-type alkaloids isolated from these plants have been reported to exhibit immunostimulatory, anti-tumor, anti-viral and anti-malarial activities [4]–[7]. For example, lycorine, a pyrrolophenanthridine alkaloid, has been demonstrated to suppress cell growth of the human leukemia cell line HL-60 [8] as well as the multiple myeloma cell line KM3 [9] by arresting the cell cycle, subsequently inducing apoptosis of tumor cells. More recently, lycorine causes a rapid turnover of protein levels of myeloid cell leukemia-1 (Mcl-1), which may play an important survival role in a variety of tumor cells including leukemia were reported [10]. So lycorine might be a good candidate therapeutic agent against leukemia. Additionally, it has also been reported that lycorine was an active component in the alkaloid portion and a good candidate for the development of new antiviral medicine in the treatment of severe acute respiratory syndrome (SARS) [11]. Galanthamine, another major Amaryllidaceae alkaloid, has also been widely used in medicine as a strong reversible inhibitor of cholinesterase to increase acetylcholine sensitivity [12]. It is a specific remedy for myasthenia gravis and poliomyelitis sequela and has also been used in the therapy of glaucoma [13] and Alzheimer’s disease [13]–[15]. Hence, galanthamine also has important medicinal value and broad application prospect [16]. At the same time, because of their several biological activities and their potential diversity in pharmacology, Amaryllidaceae alkaloids have also attracted great interest of synthetic organic chemists [17]–[25].

It is well known that the generation of large-scale expressed sequenced tags (ESTs) is a very useful approach to describe the gene expression profile and sequence of mRNA from a specific organism and stage (especially in non-model species). ESTs represent a valuable sequence resource for research and breeding, as they provide comprehensive information regarding the transcriptome [26]. They have played significant roles in functional genomics research for discovery of novel genes together with identifying different protein groups (e.g. proteins with signal peptides) other than the whole genome [27]–[29], developing SSRs and SNPs markers [30]–[34], allowing large-scale expression analysis [35], improving genome annotation [36], and elucidating phylogenetic relationships [37].

Next-generation sequencing (NGS) technologies such as the Illumina Solexa, Roche 454, and ABI SOLiD platforms have greatly decreased the cost and time required for receiving genomic and transcriptomic data [38]. By generating sufficiently long sequence reads, Roche 454 pyrosequencing using Genome Sequencing (GS) FLX technology makes it possible to compensate for the lack of a reference genome during *de novo* sequence assembly with the concurrent improvements of *de novo* assembly software [39]. Meanwhile, it is particularly useful as a shotgun method for generating EST data and a powerful method for whole genome transcriptome analysis and gene discovery with pyrosequencing of uncloned cDNAs [40]. So far, a large number of plants [26], [34], [40]–[42] including Arabidopsis [43], *Artemisia annua* [44], cucumber [45], *Medicago* [46], maize [47], barley [48] and *Jatropha curcas* (Barbados Nut) [49] have been performed for transcriptome analyses by Roche 454 pyrosequencing. Also, many EST libraries of a wide range of plant species have been constructed for genes involved in plant growth and differentiation [46], [50], biochemical pathways [47], [48], secondary metabolism [51], [52] as well as responses to environmental stresses and pathogen attack [53].

The goal of this study was to characterize the transcriptome of *L. aurea* in *Lycoris* species using high-throughput Roche 454 pyrosequencing. As one of the Amaryllidaceae plants, *L. aurea* is an indigenous and popular ornamental herb in China [54]. It is well-known not only for the high economic value in horticulture but also for the alkaloids it produces, among which galanthamine and lycorine are the major ingredients [55], [56]. In recent years, studies have reported that *L. aurea* is a good material for extraction of galantamine and other alkaloids [55], [57]. However, little research has been performed to address the Amaryllidaceae alkaloids biosynthesis-related genes (especially for galanthamine biosynthesis). Additionally, to date, there are only less than 9,000 ESTs available for *Lycoris*. And limited by the availability of genomic information, studies of *Lycoris* have mainly focused on karyotypes analysis 1,3,58,59, morphology [60], medicine [11], [13]–[15], and molecular aspect [2], [61]–[64]. Hence, determination of the genetic pathways and specific genes involved in Amaryllidaceae alkaloids biosynthesis and some other aspects of *Lycoris* could be beneficial for humans and enrich our knowledge and understanding of functional genomics and biological research. Transcriptome sequencing might provide such a useful tool. After preparing a cDNA library by pooling total RNA from various organs and tissues, including leaves with sodium nitroprusside (SNP), salicylic acid (SA), or methyl jasmonate (MeJA) treatment, stems and flowers at the bud, blooming, and wilting stages, we sequenced ESTs from this library. The transcriptome sequences were then annotated by BLASTing against public databases. Subsequently, the annotated sequences were clustered into putative functional categories using the Gene Ontology (GO) framework and grouped into pathways using the Kyoto Encyclopedia of Genes and Genomes (KEGG). This transcriptome dataset represents the first exploration of *L. aurea* and provides an invaluable new resource for functional genomics and biological research in *L. aurea*. The results described herein provide a material basis for future genetic linkage and quantitative trait loci (QTL) analysis and may serve to guide further gene express and functional genomic research in future.

## Results and Discussion

### cDNA Synthesis and Normalization

In order to achieve *L. aurea* transcriptome, total RNA was extracted from a variety of adult organs and tissues, including the leaves, stem, and flowers. It has been reported that in some plants of Amaryllidaceae family, the improved production of galanthamine was examined in MeJA-treated tissues and 1-aminocyclopropane-1-carboxylic acid (ACC)-treated somatic embryos respectively [65], [66]. And in our previous study, we also found that the content of galanthamine in *Lycoris chinensis* and *Lycoris radiata* seedlings would have been affected after treating with sodium nitroprusside (SNP), salicylic acid (SA), or MeJA [67], [68]. For the purpose of improving mRNA abundance of genes related to Amaryllidaceae alkaloids biosynthesis, the leaves were treated with those abiotic elicitors for RNA extraction. Quality of the RNA as determined by agarose gel electrophoresis and OD_260_/OD_280_ ratio (2.0 ± 0.10) was found to be suitable for cDNA synthesis. After that, equal quantities of RNA from different samples were mixed together and normalized cDNA was synthesised. It has been reported that normalization of the cDNA greatly reduces the frequency of abundant transcripts, and increases the rate recovery of unique transcripts [69]. After subjecting to quality control experiment, the normalized cDNA was used to construct a cDNA library. Then the library was sequenced by a Roche 454 GS FLX.

### 454 Pyrosequencing using GS FLX Titanium Platform and Reads Assembly

**Figure 1:**
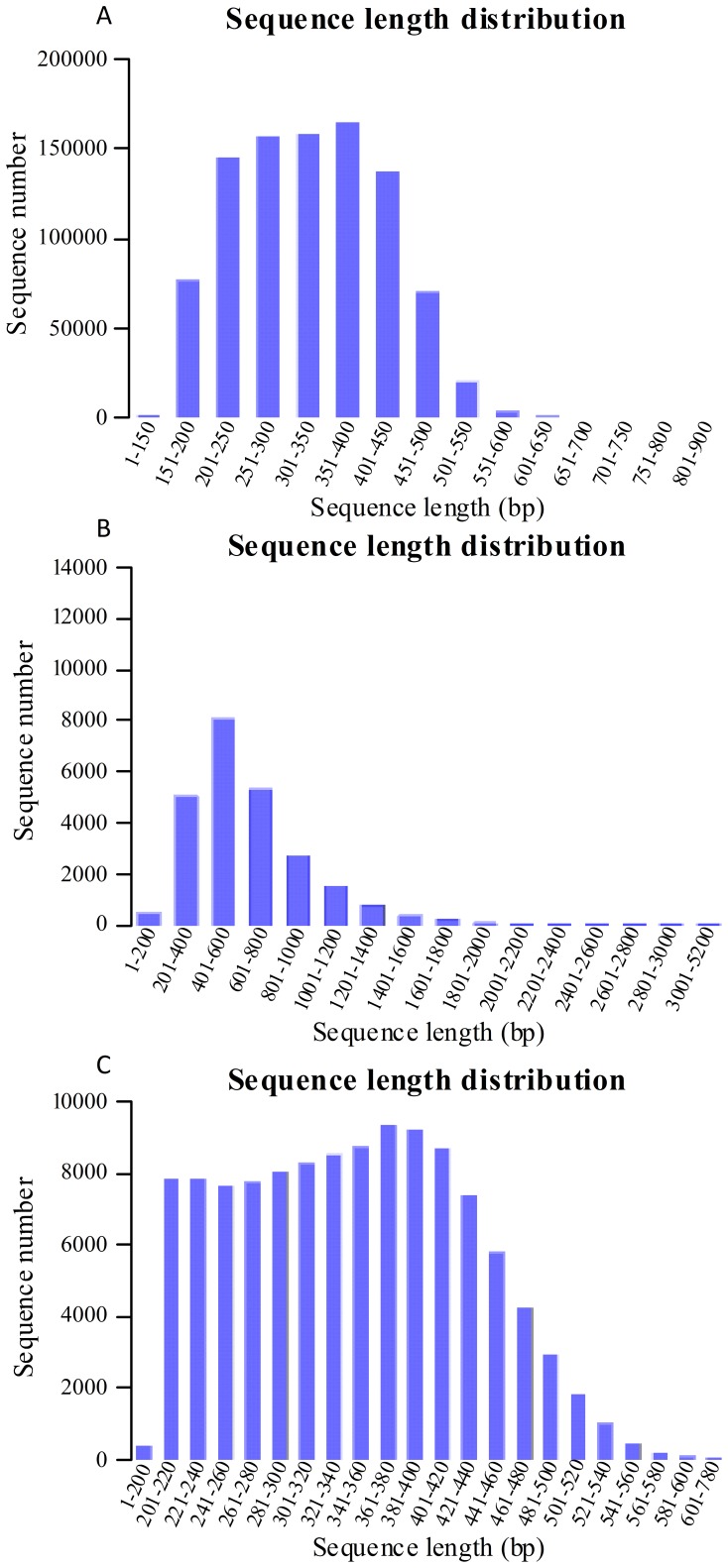
Length distribution of *L. aurea* transcriptomic ESTs. (A) total transcriptomic reads, (B) contigs, (C) singletons.

One-plate 454 pyrosequencing reaction of the normalized cDNA was done using GS FLX titanium platform. The reads produced by the Roche 454 GS FLX were used for clustering and *de novo* assembly. After eliminating primer and adapter sequences and filtering out the low-quality reads, a total of 937,990 high-quality transcriptomic raw sequence reads with a total size of 308,633,593 bp were obtained. Size distribution of these reads is shown in Figure 1A. Length of these reads ranged between 150 and 854 bases with an average length of 329 bp per read (Figure 1A). Clustering and assembly of these raw reads was done using GS *de novo* assembler [70], [71]. This assembler can assemble the data under genomic or cDNA option. After clustering and assembly, a non-redundant set of 141,111 expressed sequence tags (ESTs), comprising 24,604 contigs and 116,507 singletons, respectively (Table 1) were obtained. Most of these contigs (95.04%) were distributed in the 200∼1,400 bp region (Figure 1B). And most of these singletons fell between 161 and 500 bp in length (Figure 1C). So far, the number of ESTs that are available from *Lycoris* is less than 9,000. Recently, by sequencing clones from three non-normalized cDNA libraries, 32,521 EST sequences were obtained and most of them were used for floral transcription factors prediction from *Lycoris longituba* [64]. Therefore, this transcriptome dataset provides a useful resource for future analyses of genes related to Amaryllidaceae alkaloids synthesis. To the best of our knowledge, this is the first comprehensive study of the transcriptome of *L. aurea*.

**Table 1:** Summary of Roche 454 GS FLX assembly and analysis of *L. aurea* transcriptomic sequences.

Dataset name	All
transcriptomic reads	937,990
total number of bases	308,633,593 bp
average read length	329 bp
No. of contigs	24,604
No. of singletons	116,507

### GC Content and Alternative Splicing

The GC content (ratio of guanine and cytosine) of all unique sequences of *L. aurea* was determined. The content of GC was 40.42% and 39.58% in contigs and singletons, respectively, giving rise to an overall GC content of 40.03%, indicating a low GC content in the cDNA of *L. aurea*. The *L. aurea* contigs were further assembled into 16,828 isogroups (all the splice variants of individual transcripts). More contigs than isogroups were found because some contigs (called isotigs, 24,463) are attributed to the same isogroups due to alternative splicing. A large number of alternative splicing could improve the utilization rate of the encoding genes. Alternative splicing is an important mechanism for regulating gene expression in eukaryotic cells, and it contributes to protein diversity.

### Functional Annotation of the Transcriptome of *L. aurea*

Similarity search for all the unique sequences was done against GenBank non-redundant protein sequences database (nr) using BLASTx. A total of 66,197 (46.91%) *L. aurea* unigenes including 18,397 contigs and 47,800 singletons were significantly matched to known genes in the public databases (with an e-value of 10^−6^) (Table S1), representing putative functional identifications for almost half of the assembled sequences. Previous studies have shown that approximately 87% of Arabidopsis 454-derived ESTs could be aligned to predicted genes [43], while 72% could be similarly identified in cucumber [45] and 54.9% in bamboo [50]. As such, our results succeeded in assigning putative identification to a significant proportion of the discovered *L. aurea* transcripts given the lack of genomic information for this species.

Amongst the unique sequences derived from contigs and singletons, coding sequences with homology to ‘NADH dehydrogenase’, ‘cytochrome c oxidase’, ‘ATP synthase’, ‘splicing factor’, ‘cytochrome P450’, ‘ubiquitin-protein ligase’, and ‘zinc finger protein’ were the most abundant. Although our research mainly focused on finding putative genes related to Amaryllidaceae alkaloids synthesis, other putative functional transcripts identified here could provide a foundation for future investigations of the roles of stress response, reproduction and defense reaction. The transcriptomic findings could also be the best source for deciphering the putative functions of novel genes, but further studies would need to be conducted to understand their molecular functions.

### GO Assignments

GO provides a structured and controlled vocabulary for describing gene products in three categories: molecular function, biological process and cellular component [72]. We added GO terms using Blast2GO [73], which is based on the automated annotation of each unigene using BLAST results against the GenBank non redundant protein database (nr) from NCBI. According to the database, a total of 36,188 unigenes could be assigned to one or more ontologies based on their similarity to sequences with previously known functions, including 43,970 sequences assigned to the molecular function category, 72,628 to the biological process category and 79,853 to the cellular component category. The assigned sequences were divided into 58 functional terms (Table S2). Because several of the sequences were assigned to more than one GO term, the total number of GO terms obtained in our dataset was bigger than the total number of the unique sequences. In total, 196, 451 GO terms were retrieved, 22.38%, 40.65% and 36.97% in the molecular function, in the cellular component and in the biological process category, respectively.

**Figure 2:**
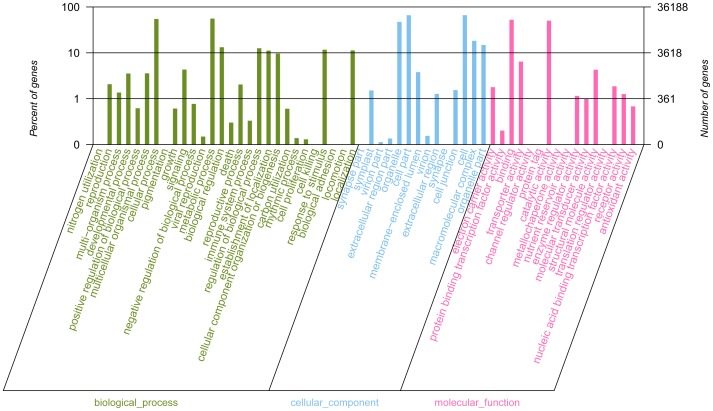
Gene Ontology (GO) terms for the transcriptomic sequences of *L. aurea*.

We used the GO annotations to assign each unigene to a set of GO Slims of the three categories, which are a list of GO terms providing a broad overview of the ontology content. A summary with the number and percentage of unigenes annotated in each GO slim term is shown (Figure 2). GO annotations for the unigenes showed fairly consistent sampling of functional classes. In the molecular function category, ‘binding’, ‘catalytic activity’, ‘transporter activity’ and ‘structural molecule activity’ comprised the largest proportion, accounting for 93.35% of the total. Whilst the cellular component category showed that many unique sequences were to likely possess ‘cell’ (29.88%), ‘cell part’ (29.88%) and ‘organelle’ (21.38%) functions. Moreover, ‘metabolic processes’ (27.75%) and ‘cellular process’ (27.29%) were among the most highly represented groups under biological functions category. This might be indicating the analyzed tissues were undergoing rapid growth and extensive metabolic activities. Genes involved in other important biological processes such as biological regulation (6.59%), regulation of biological process (6.27%) and response to stimulus (5.83%) were also identified (Figure 2). In summary, these terms account for a large fraction of the overall assignments in *L. aurea* transcriptomic dataset. Understandably, genes encoding these functions may be more conserved across different species and are thus easier to annotate in the database.

### COG Annotation

**Figure 3:**
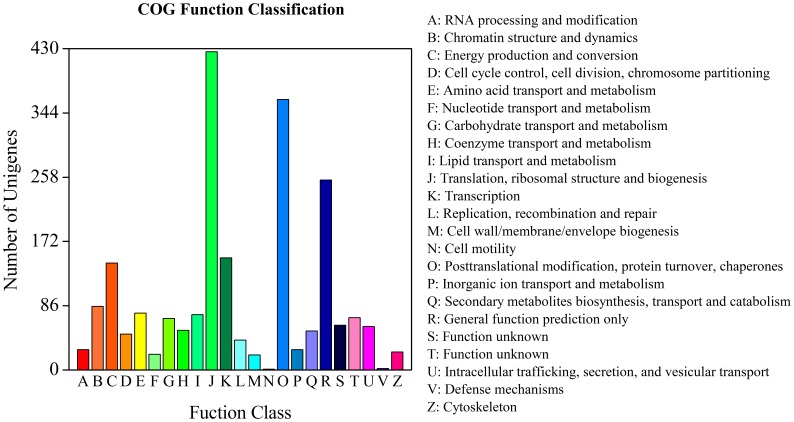
Clusters of orthologous groups (COG) classifications of unique *L. aurea* sequences.

Assignments of COG were used to predict and classify possible functions of the unique sequences. Based on sequence homology, 2,142 unique sequences had a COG functional classification. These sequences were classified into 23 COG categories (Figure 3). ‘Translation, ribosomal structure and biogenesis’ represented the most common category (426, 19.89%), followed by ‘posttranslational modification, protein turnover, chaperones’ (362, 16.90%) and ‘General function prediction only’ (254, 11.86%). ‘Cell motility’ (1, 0.05%), ‘defense mechanisms’ (2, 0.09%) and ‘Cell wall/membrane/envelope biogenesis’ (7, 0.93%) were the smallest COG categories.

### KEGG Pathway Mapping

**Figure 4:**
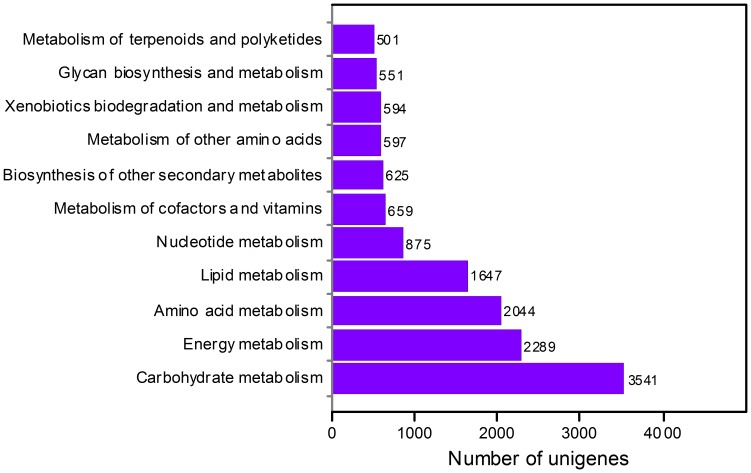
KEGG metabolism pathway categories assigned with *L. aurea* unigenes.

Besides GO analysis, KEGG [74] pathway mapping based on enzyme commission numbers for assignments was also carried out for the assembled sequences, which is an alternative approach to categorize genes functions with the emphasis on biochemical pathways. Ortholog assignment and mapping of the contigs and singletons to the biological pathways were performed using KEGG automatic annotation server (KAAS). According to the KEGG results, 21,274 *L. aurea* unigenes comprising 7,097 contigs and 14,177 singletons were mapped onto a total of 295 predicted metabolic pathways, representing compound biosynthesis, degradation, utilization and metabolism (Table S3). It also assigned EC numbers for 3,222 contigs and singletons, and they were mapped to respective pathways. Transcripts identified as related to the following global map or cellular processes were the most abundant: metabolic pathways (6,048 unigenes), biosynthesis of secondary metabolites (2,606), ribosome (1,444), microbial metabolism in diverse environments (1,305) and protein processing in endoplasmic reticulum (793). The largest category was metabolism (13,923) which included carbohydrate metabolism (3,541), energy metabolism (2,289), amino acid metabolism (2,044), lipid metabolism (1,647), nucleotide metabolism (875), metabolism of cofactors and vitamins (659), biosynthesis of other secondary metabolites (625) and other subcategories (Figure 4). In the secondary metabolism category, the most represented subcategories were phenylpropanoid biosynthesis (226), terpenoid backbone biosynthesis (161), tropane, piperidine and pyridine alkaloid biosynthesis (112), metabolism of xenobiotics by cytochrome P450 (102), carotenoid biosynthesis (99), limonene and pinene degradation (96), flavonoid biosynthesis (84), stilbenoid, diarylheptanoid and gingerol biosynthesis (76), and chloroalkane and chloroalkene degradation (69) was also classified. In addition to metabolism pathways, genetic information processing genes (6,850) were highly represented categories. Transcription, sorting and degradation, replication and repair, folding, and translation were included in these categories.

KEGG pathway analysis and COG analysis are helpful for predicting potential genes and their functions at a whole transcriptome level. The predicted metabolic pathways, together with the COG analysis, are useful for further investigations of gene function in future studies.

### Possible Genes Related to Amaryllidaceae-type Alkaloids Biosynthesis

The transcriptome of *L. aurea* was primarily examined to identify a wide range of candidate genes that might be functionally associated with Amaryllidaceae alkaloids biosynthesis. Since the isolation of the first alkaloid, lycorine, from *Narcissus pseudonarcissus* in 1877, substantial progress has been made in examining the Amaryllidaceae plants, although they still remain a relatively untapped phytochemical source. At present, over 100 alkaloids have been isolated from different Amaryllidaceae plants [75], although their structures vary considerably, these alkaloids are considered to be biogenetically related. Mainly, the large numbers of structurally diverse Amaryllidaceae alkaloids are classified into 9 skeleton types, for which the representative alkaloids are: norbelladine, lycorine, homolycorine, crinine, haemanthamine, arciclasine, tazettine, montanine and galanthamine.

Most of the biosynthetic research done on Amaryllidaceae alkaloids was carried out in 1960s and 1970s. Since then, studies have been reported that the biosynthesis of Amaryllidaceae alkaloids belongs to different ring type subgroups [75]–[78]. And the noteworthy study could be the biosynthesis of galanthamine and related alkaloids [76]. For example, it has been considered that L-phenylalanine (L-phe) and L-tyrosine (L-tyr) would be the precursors of Amaryllidaceae alkaloids biosynthesis. Although L-phe and L-tyr are closely related in chemical structure, they are not interchangeable in plants. The presence of the enzyme phenylalanine ammonia-lyase (PAL) has been demonstrated in Amaryllidaceae plants [68], [79] and the elimination of ammonia mediated by this enzyme is known to occur in an antiperiplanar manner to give *trans*-cinnamic acid, with loss of the β-*pro*-S hydrogen [80]. Besides, it has been proposed that Amaryllidaceae alkaloids could be regarded as derivatives of the key intermediate 4′-*O*-methylnorbelladine [77]. There are three different groups of Amaryllidaceae alkaloids that are biosynthesized by three modes of intramolecular oxidative phenol coupling (*para-para′*, *ortho-para′* and *para-para′*) [75], [76], [78].

Moreover, plant cytochromes P450 (P450s), as one of the biggest gene superfamilies in plant genomes, might also be involved in the Amaryllidaceae alkaloids biosynthesis. It has been well-known that P450s catalyze a wide variety of monooxygenation/hydroxylation reactions in primary and secondary metabolism. They participate in a variety of biochemical pathways to produce primary and secondary metabolites such as phenylpropanoids, alkaloids, terpenoids, lipids, cyanogenic glycosides, and glucosinolates, as well as plant hormones [81]–[84]. For example, in some kinds of plants, several P450s in the CYP80 and CYP719 families, known to catalyze reactions (such as C-O and C-C phenol-coupling reaction) atypical for P450s, function in Benzylisoquinoline alkaloids (BIAs) biosynthesis [85]–[88]. Although little is known about the relationship between P450s and Amaryllidaceae alkaloids biosynthesis, it could also be postulated that P450s might catalyze the stereospecific reactions in some steps of Amaryllidaceae alkaloids biosynthesis pathways. Additionally, O-methyltransferase (*OMT*) acts as an important enzyme could also have participated in the galanthamine biosynthesis [76].

According to our sequence analysis and published literatures, many genes might be involved in Amaryllidaceae alkaloids synthesis, including phenylalanine ammonia-lyase (*PAL*), tyrosine decarboxylase (*TYDC*), *OMT*, *P450s*, N-Methyltransferase (*NMT*), and other potential candidates were identified (Table 2). For example, 26 unique sequences were identified as *PAL1*, *PAL2*, and *PAL3* with similarities ranging from 62%∼100%, respectively. 91 unique sequences were identified as *OMT* with similarities ranging from 51%∼100%, respectively. Additionally, only 6 unique sequences were identified as *TYDC* with similarities ranging from 55%∼88%, respectively. To the best of our knowledge, these putative *TYDC* genes are first reported in *L. aurea*.

**Table 2:** Selected genes of interest for Amaryllidaceae-type alkaloids biosynthesis in the *L. aurea* transcriptome, including the contigs and singletons.

Candidate genes	Hit(s)	Similarity (%)	Length (bp)
*PAL*	25	62∼100	215∼560
*NMT*	191	76∼100	213∼1125
*P450*	214	55∼100	221∼1733
*OMT*	91	51∼100	246∼1385
*TYDC*	6	55∼88	299∼834

### SSR and SNP Discovery

SSRs, or microsatellites, are neutral molecular markers that widely distribute in a genome. They consist of repeated core sequences of 2∼6 base pairs in length. Among the various molecular markers, SSRs have been proven to be an efficient tool for performing QTL analysis, constructing genetic linkage and evaluating the level of genetic variation in a species because of the high diversity, abundance, neutrality and co-dominance of microsatellite DNA [31]–[33].

**Figure 5:**
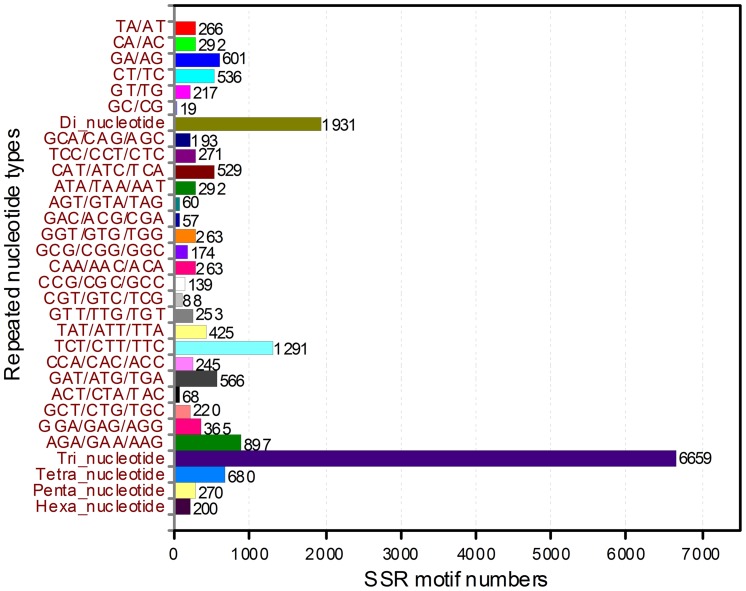
Distribution of simple sequence repeats (SSR) among different nucleotide types found in the transcriptome of *L. aurea.*

In total, 9,740 SSRs were obtained from the transcriptomic dataset. Of these, the most frequent repeat motifs were tri-nucleotides, which accounted for 68.37% of all SSRs, followed by di-nucleotide repeats (19.83%), tetranucleotides (6.98%), pentanucleotides (2.77%), and hexanucleotides (2.05%) (Figure 5). Based on the distribution of SSR motifs, (GA/AG)_n_, (CT/TC)_n_ and (CA/AC)_n_ were the three predominant types among the di-nucleotide repeats motifs, with frequencies of 31.12%, 27.76% and 15.12%, respectively. In the 20 types of tri-nucleotide repeats, CTT (19.39%) was the most common motif, followed by AAG (13.47%), GAT (8.50%) and ATC (7.94%). To date, only a few microsatellites have been available for *L. aurea* from NCBI. Thus, the development of SSRs for this species is highly desirable.

**Figure 6:**
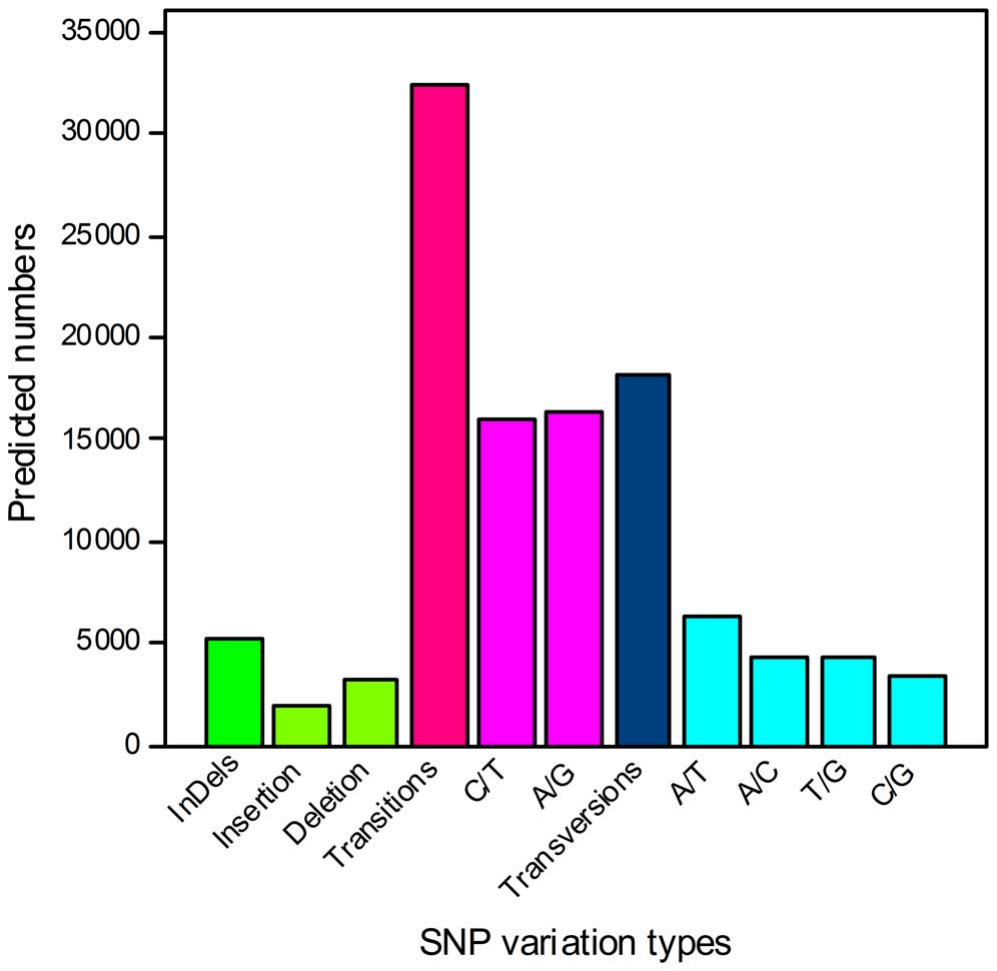
Distribution of putative single nucleotide polymorphisms (SNP) in the transcriptome of *L. aurea*.

SNPs were identified from alignments of multiple sequences used for contig assembly. By excluding those that had mutation frequency of bases lower than 1%, we obtained a total of 55,800 SNPs, of which 5,160 were putative indels (In), 32,440 were putative transitions (Ts) and 18,220 were putative transversions (Tv), giving a mean In: Ts: Tv ratio of 1∶6.29∶3.53 across the transcriptome of *L. aurea* (Figure 6). The AG/GA, CT/TC and AT/TA SNP types were the most common. In contrast, GC/CG types were the smallest SNP types because of the differences in the base structure and the number of hydrogen bonds between different bases. Multiple sequence alignment also identified a total of 5,160 indels across the transcriptome. It should be treated with caution because of technical problems associated with Roche 454 GS FLX pyrosequencing [42].

### Conclusions

In this study, *de novo* transcriptome sequencing for *L. aurea* using the 454 GS FLX was performed for the first time. A total of 937,990 high-quality transcriptomic reads were obtained, giving rise to an average of 329 bp per read. A significant number of putative metabolic pathways and functions associated with the unique sequences were identified. Moreover, a large number of SNPs and SSRs were predicted and can be used for subsequent marker development, genetic linkage and QTL analysis. Many candidate genes that are potentially involved in Amaryllidaceae alkaloids synthesis were identified for the first time and are worthy of further investigation. Our study provides the largest number of ESTs to date and lays the initial groundwork for indepth, functional transcriptomic profiling of *L. aurea*.

## Materials and Methods

### Plant Material

*L. aurea* used in this study were collected from Institute of Botany, Jiangsu Province & Chinese Academy of Sciences, Nanjing, China. In order to achieve *L. aurea* transcriptome, samples were collected from a variety of adult organs and tissues, including the stem, flowers, and leaves. The stem and flowers collected for the RNA extraction were at their bud, blooming, and wilting stages respectively. For the leaves collection, the seedlings grown in illuminating incubator (25 ± 1°C ; 14/10 h photoperiod) were treated with 500 µM sodium nitroprusside (SNP), 250 µM salicylic acid (SA), or 100 µM methyl jasmonate (MeJA) for 1, 6, 12, 24, and 48 h. At above indicated time point of treatment, the samples were harvested. All of the samples were immediately frozen in liquid nitrogen and stored at –80°C until use.

### RNA Extraction, cDNA Library Construction and 454 Sequencing

Total RNA was extracted from these materials using TRIzol Reagent (Invitrogen, USA) according to the manufacturer’s instructions. The quality of total RNA was determined using a NanoDrop spectrophotometer (Thermo, USA) and RNA samples with a 260 of 280 ratio from 1.9 to 2.1were selected for the next analysis. After that, equal quantities of total RNA from each sample (∼0.35 mg total RNA) were mixed together and delivered it to Shanghai Majorbio Bio-pharm Biotechnology Co., Ltd. (Shanghai, China) for the construction of the cDNA library.

The cDNA library was constructed using the Creator^TM^ SMART^TM^ cDNA library construction kit (Clontech Laboratories Inc., Mountain View, CA, USA) and following the manufacturer’s protocol step-by-step. With agarose gel electrophoresis and extraction of DNA from gels, DNA bands (500∼800 bp) were purified, blunt ended followed by ligation with adapters and finally immobilized on beads. The quality control of a double DNA library was performed using High Sensitivity Chip (Agilent Technologies). The concentration and the proper ligation of the adapters were examined by using TBS 380 Fluorometer. After the examination, one-plate, whole-run sequencing was performed on Roche 454 GS FLX Titanium chemistry (Roche Diagnostics, Indianapolis, IN, USA) by Shanghai Majorbio Bio-pharm Biotechnology Co., Ltd. following the manufacturer’s protocol.

### Sequence Cleaning and Assembly

The initial assembly comprised 937,990 reads. For each sequence, low-quality bases and the sequencing adapter were trimmed using LUCY (http://lucy.sourceforge.net/) and SeqClean (http://compbio.dfci.harvard.edu). The remained sequencing reads were assembled using the Newbler software package (a *de novo* sequence assembly software) with the “extend low depth overlaps” parameter. All of the ESTs from the Roche 454 were used to run the final assembly of *L. aurea*.

### Functional Annotation with BLAST Program

BLASTx searches [89] of the GenBank nr database hosted by NCBI (http://www.ncbi.nlm.nih.gov/) were performed on all unique sequences to identify the putative mRNA functions. Additionally, GO terms (http://www.geneontology.org) were extracted from the best hits obtained from the BLASTx against the nr database using Blast2GO. These results were then sorted by GO categories using in-house Perl scripts. BLASTx was also used to align unique sequences to the Swiss-Prot database (http://web.expasy.org/docs/swiss-prot_guideline.html), Kyoto Encyclopedia of Genes and Genomes (KEGG) and Clusters of Orthologous Groups (COG) (http://www.ncbi.nlm.nih.gov/COG/) (with the e-value of 10^−6^) to predict possible functional classifications and molecular pathways [90], [91].

### Identification of EST-SSR Motifs and EST-SNPs

The unique sequences were screened for microsatellites using software Mreps (http://bioinfo.lifl.fr/mreps/) with default parameters. Perfect di-, tri-, tetra-, penta-, and hexa-nucleotide motifs were detected, and all SSR types required a minimum of 6 repeats. Potential SNPs were extracted using VarScan (http://varscan.sourceforge.net) with the default parameter only when both alleles were detected from 454 reads. Since no reference sequences were available, SNPs were identified as superimposed nucleotide peaks where two or more reads contained polymorphisms at the variant allele.

### Data Deposition

The Roche 454 reads of *L. aurea* were submitted to NCBI Sequence Read Archive under the accession number of SRP018374.

## Supporting Information

Table S1 Summary of BLASTx results for contigs and singletons of *L. aurea*.
(XLS)

Table S2 Categories of Gene Ontology of *L. aurea* unique sequences.
(XLS)

Table S3 KEGG summary of *L. aurea* unique sequences.
(XLS)
